# Catalytic asymmetric carbenoid α-C–H insertion of ether

**DOI:** 10.1039/d4ra02206h

**Published:** 2024-05-13

**Authors:** Xin Li, San-Hong Yue, Zi-Yang Tan, Shu-Bo Liu, De-Xiang Luo, Ying-Jun Zhou, Xiao-Wei Liang

**Affiliations:** a Xiangya School of Pharmaceutical Sciences, Central South University Changsha 410013 China zhouyingjun@csu.edu.cn; b Hunan Key Laboratory of Skin Cancer and Psoriasis, Xiangya Hospital, Central South University Changsha 410013 China xiaowei.liang@csu.edu.cn

## Abstract

Significant advancements have been made in catalytic asymmetric α-C–H bond functionalization of ethers *via* carbenoid insertion over the past decade. Effective asymmetric catalytic systems, featuring a range of chiral metal catalysts, have been established for the enantioselective synthesis of diverse ether substrates. This has led to the generation of various enantioenriched, highly functionalized oxygen-containing structural motifs, facilitating their application in the asymmetric synthesis of bioactive natural products.

## Abstract 2

Significant advancements have been made in catalytic asymmetric α-C–H bond functionalization of ethers *via* carbenoid insertion over the past decade.

## Graphical abstract

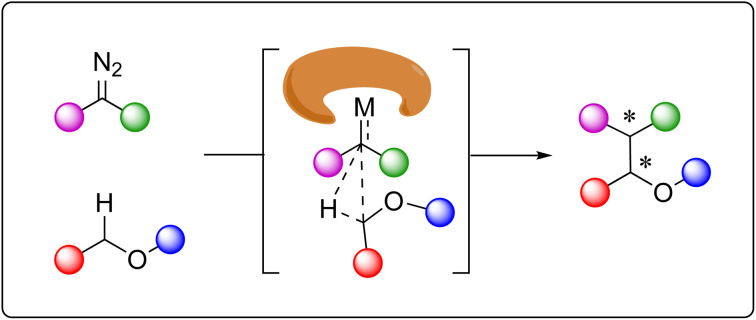

## 1. Introduction

**Fig. 1:**
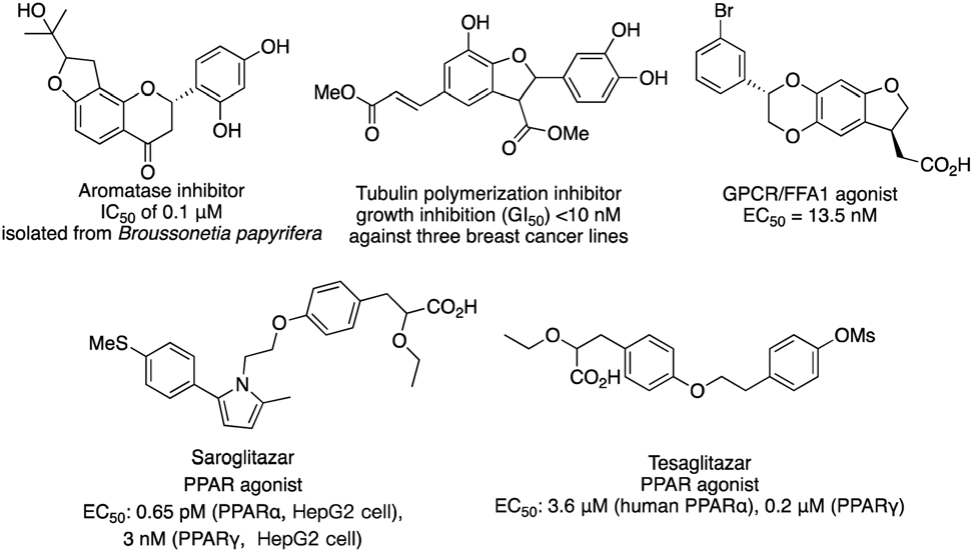
Representative bioactive nature products.

**Fig. 2:**
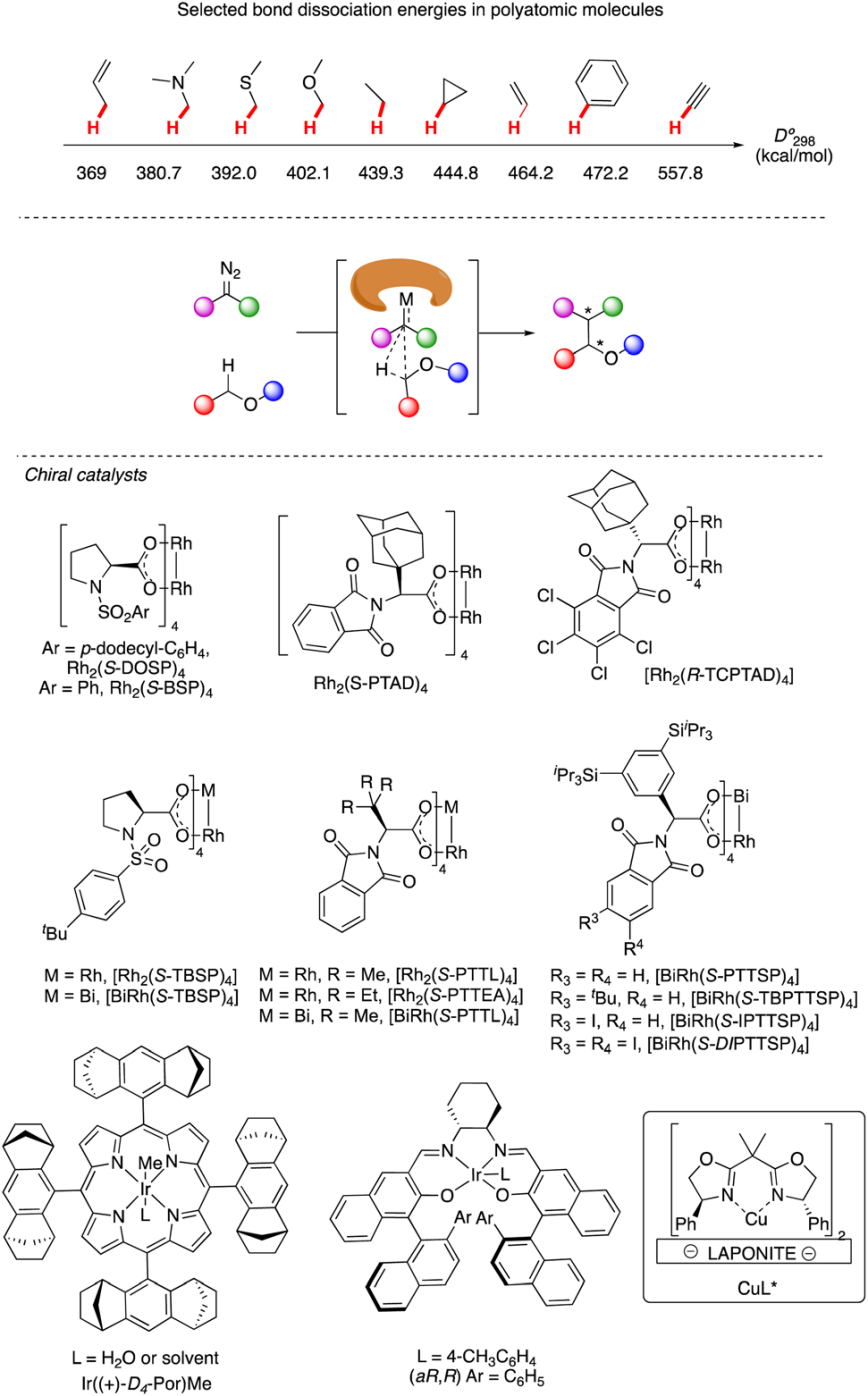
Representative catalysts for α-C–H bond carbenoid insertion of ether.

C–H functionalization continues to be one of the forefronts of organic chemistry.^1^ The resurgence of carbenoid transformations from diazo compounds has garnered significant attention due to their effectiveness in transition-metal and enzyme-catalyzed C–H functionalization.^2^ While the spontaneous decomposition of ethyl diazoacetate was first studied in 1906 by Silberrad and Roy,^3^ it was not until Yates suggested the participation of transition metals in diazo decompositions that the true potential of diazo chemistry began to emerge.^4^ Particularly noteworthy is the ability of *in situ* formed highly active metal carbenoids to insert into C–H bonds of aromatics and alkanes.^5^ Especially with direct C–H bond functionalization with different transition metals, such as Cu,^6^ Ir,^7^ Rh^8^ and others,^9^ the *in situ* formed highly active metal carbenoids are ideal to insert into C–H bonds of aromatics and alkanes. Despite extensive research on alkyl amines and alcohols, catalytic asymmetric α-C–H functionalization of ethers remains challenging. Early studies by Adams, Frenette, and coworkers in 1989 demonstrated that the C–H bond adjacent to an ether is a preferred site for insertion catalyzed by rhodium acetate.^10^ Subsequent breakthroughs by Doyle and Davies and their coworkers showcased the remarkable efficiency and enantioselectivity achievable using chiral rhodium carboxamides (Rh_2_(5*S*-MEPY)_4_) and newly designed dirhodium complexes, respectively.^11^ Since then, the chiral dirhodium complex has dominated the asymmetric C–H insertion of diazo compounds.^12^Fig. 1 showcases highly active ether natural products and drug candidates, emphasizing the significance of ether compound synthesis methodology in drug discovery and natural product synthesis (Fig. 1). Ether compounds possess higher α-C–H bond dissociation energy and limited metal coordination capability compared to compounds such as amines, sulfur, and olefins. This characteristic presents a challenge for the functionalization of α-C–H bonds in ethers (Fig. 2). Herein, we present a comprehensive review of recent advancements in α-C–H bond carbenoid insertion of ethers, underscoring the importance of this methodology in the total synthesis of ether natural products.^13^

## 2. Intramolecular α-C–H bond carbenoid insertion of ether

### 2.1 Synthesis of dihydrobenzofurans

**Scheme 1:**
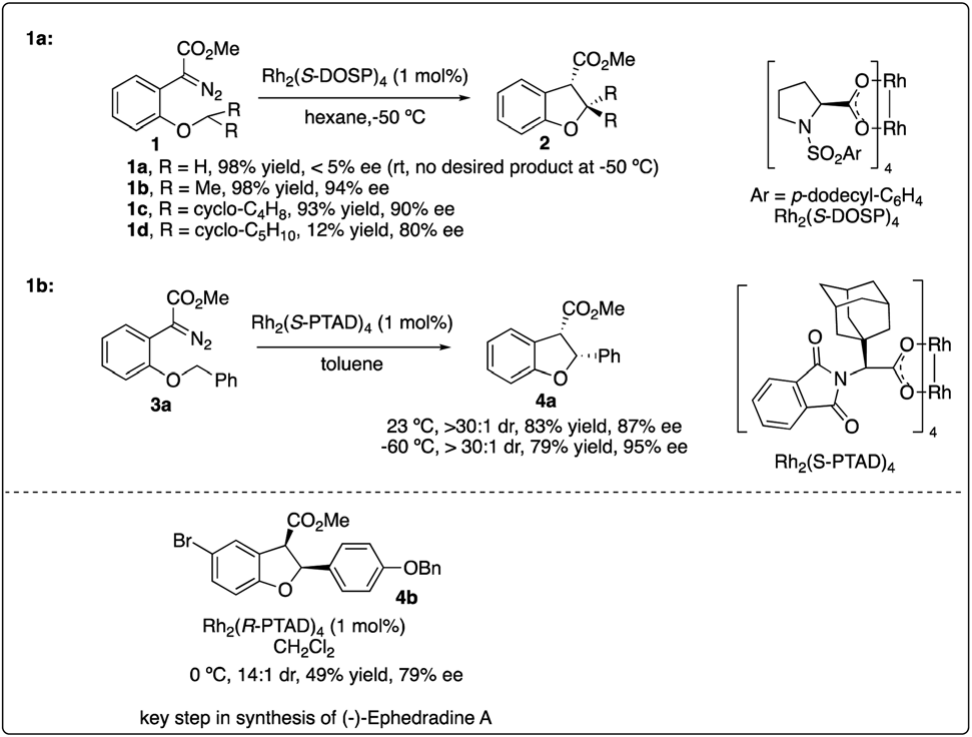
Enantioselectivity intramolecular C–H insertion reactions reported by Davises group.

In 1996, Davies and his colleagues reported the development of a newly designed chiral catalyst, Rh_2_(*S*-DOSP)_4_, commonly known as Davies's catalyst, and successfully applied it to intermolecular enantioselective C–H insertion reactions.^11*b*^ Subsequently, they achieved the intramolecular asymmetric synthesis of dihydrobenzofuran 2 with high enantioselectivity using the same robust catalyst (Scheme 1a).^14^ Through the exploration of intramolecular substrates, they investigated the selectivity between tertiary and secondary sites, highlighting the importance of both the site of C–H insertion and the catalyst in facilitating effective asymmetric intramolecular C–H insertion reactions.

In 2006, Davies and colleagues^15*a*^ introduced a novel adamantly variant, Rh_2_(*S*-PTAD)_4_, as a backup chiral catalyst to address instances where Rh_2_(*S*-DOSP)_4_ exhibited low asymmetric induction. They successfully demonstrated the enhanced ability of Rh_2_(*S*-PTAD)_4_ in promoting enantioselective intramolecular C–H insertion of α-phenyldiazoacetate 3a, serving as a pivotal step in the synthesis of (−)-ephedradine without the need for a chiral auxiliary (Scheme 1b). Furthermore, Davies and Walji developed an immobilization strategy using Argopore resin to heterogenize chiral rhodium catalysts, enabling their application in asymmetric intramolecular insertion reactions.^15*b*^ This strategy induced similar reactivity and stereoselectivity as their homogeneous counterparts, facilitating the recycling of rhodium catalysis without compromising catalytic efficiency or stereoselectivity.

**Scheme 2:**
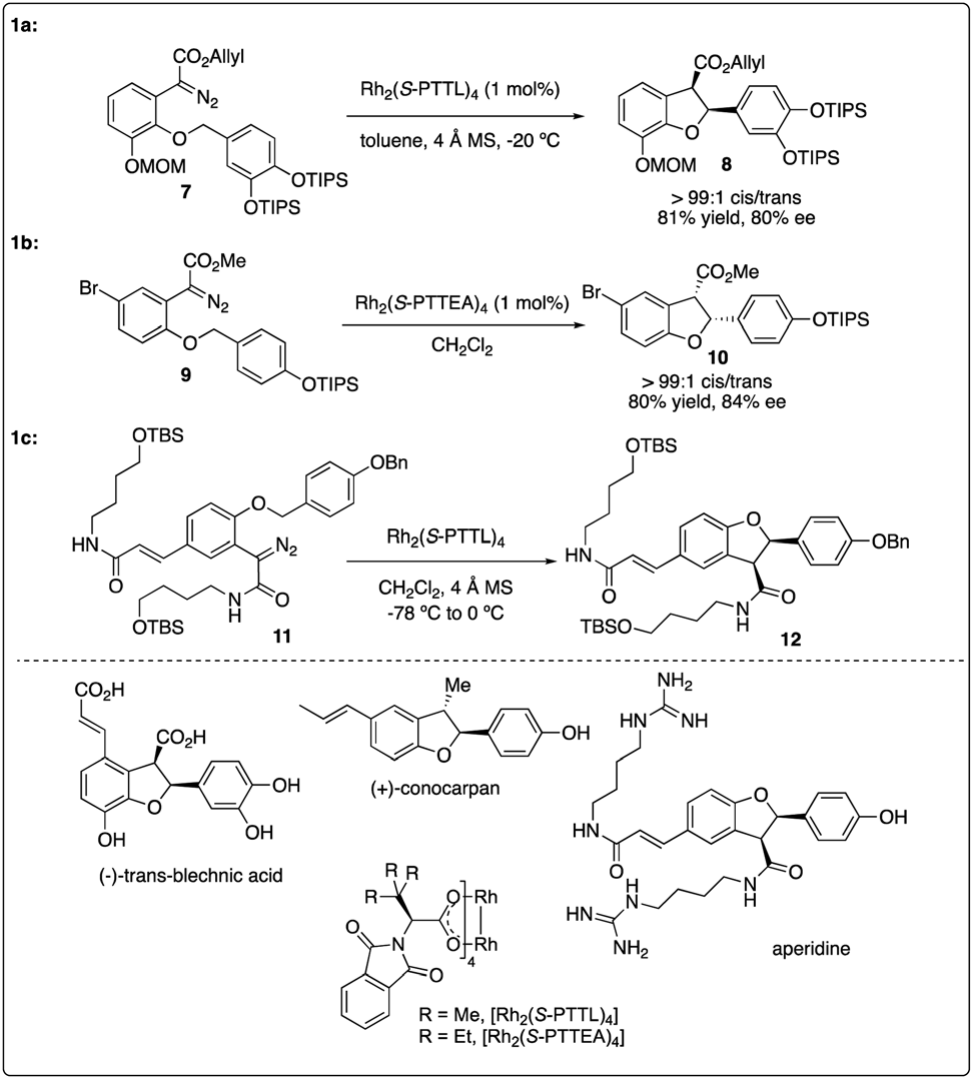
Enantioselectivity intramolecular C–H insertion reactions reported by Hashimoto group.

**Scheme 3:**
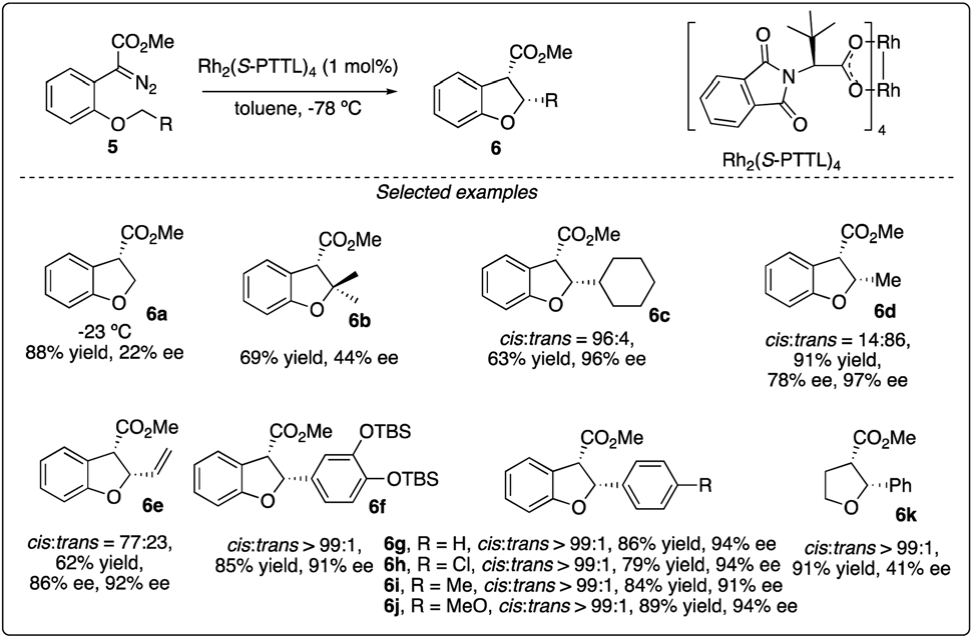
Key step in natural product total synthesis reported by Hashimoto group and Wakimoto group.

Chiral dirhodium carboxamide (*e.g.*, Rh_2_(5*S*-MEPY)_4_) and carboxylate (such as Rh_2_(*S*-DOSP)_4_) catalysts have been demonstrated as efficient catalysts to construct optically active molecules by the enantioselective intramolecular C–H insertion reactions. Subsequently, Hashimoto and colleagues^16^ identified Rh_2_(*S*-PTTL)_4_ (referred to as Hashimoto's catalyst) as the optimal catalysis for the enantioselective C–H insertion reaction of α-phenyldiazoacetate 5 (Scheme 2). Substrates bearing with a benzene ring consistently yielded excellent *cis*/*trans* ratios and high ee values (6f–6g), while those with simple methine or methyl groups resulted in disappointment outcomes (6a–d). Notably, the product containing a 3,4-(TBSO)_2_C_6_H_3_ group (6f) features an interesting motif found in dihydrobenzofuran-type neolignan blechnic acid. This motif was exploited in their following work on the asymmetric total synthesis of (−)-*trans*-blechnic acid, wherein they modified the rhodium catalyzed insertion reaction reported earlier (Scheme 3a).^17^ Additionally, aliphatic substrates were tested, yielding excellent diastereoselectivity, high yields and moderate enantioselectivity under standard reaction conditions (6k).

In 2009, Hashimoto and colleagues achieved the asymmetric synthesis of (+)-conocarpan with excellent diastereoselectivity and good enantioselectivity by employing Rh_2_(*S*-PTTEA)_4_-catalyzed intramolecular C–H insertion reaction of diazo ester 9 as the key step (Scheme 3b).^18^ 2011, Wakimoto and coworkers reported the total synthesis of aperidine, wherein the key carbenoid insertion step was catalyzed Hashimoto's catalyst Rh_2_(*S*-PTTL)_4_, affording the product in 75% yield and completely stereoselective manner (Scheme 3c).^19^

**Scheme 4:**
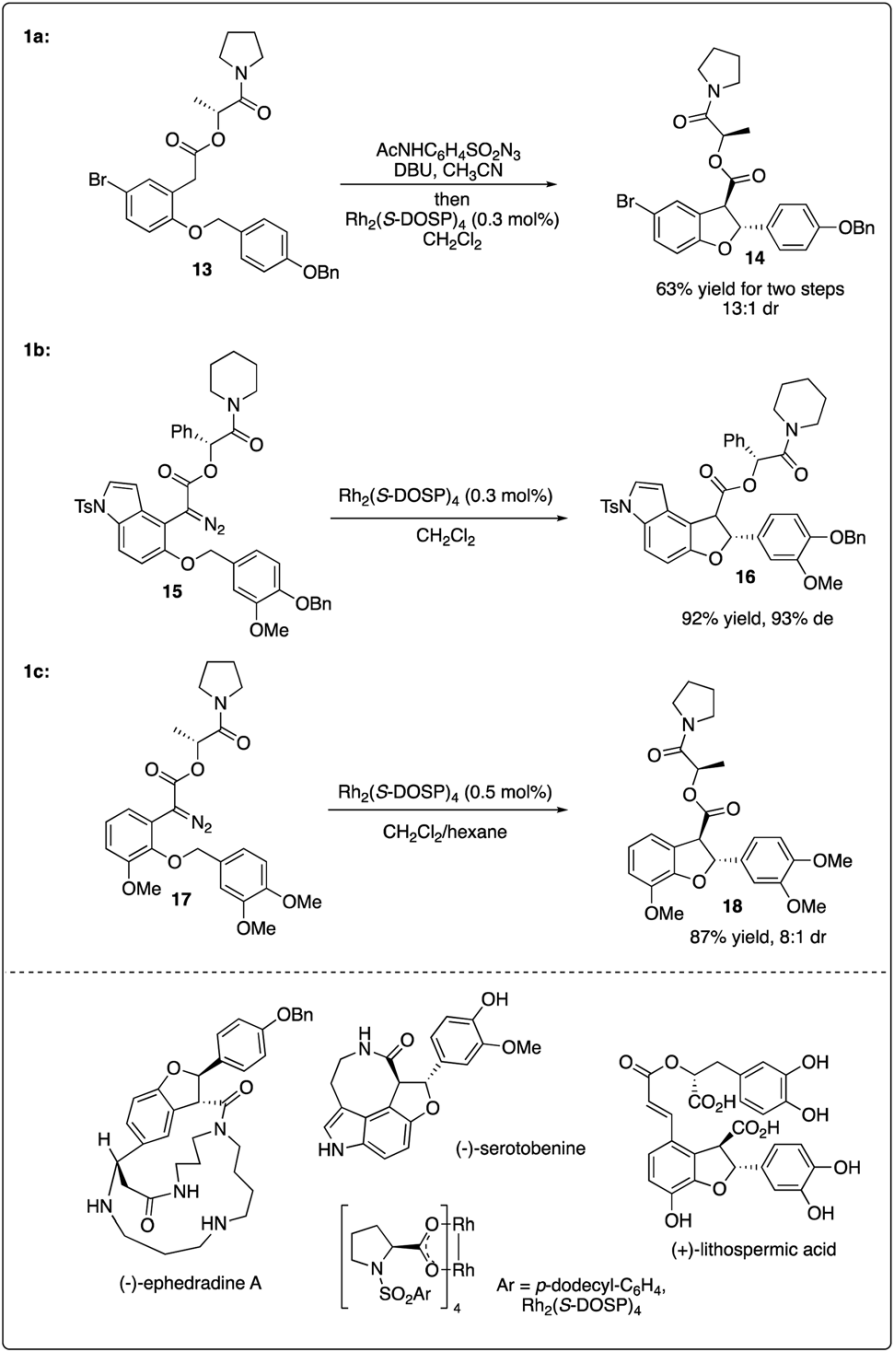
Key steps in natural product total synthesis combining with chiral auxiliary and chiral rhodium strategy.

Sometimes, the use of a chiral auxiliary alone may not suffice to achieve the necessary asymmetric induction in rhodium-catalyzed C–H carbenoid insertion reactions. To achieve good enantioselectivity in the synthesis of nature products, double asymmetric induction is frequently employed. Fukuyama and colleagues reported the Rh_2_(*S*-DOSP)_4_-catalyzed key enantioselective synthesis step of (−)-ephedradine A by utilizing intramolecular C–H insertion of diazoacetate bearing a chiral auxiliary, resulting in the formation of a *trans*-dihydrobenzofuran with excellent diastereoselectivity (Scheme 4a).^20^ Subsequently, they employed a similar strategy combining a chiral rhodium catalyst and chiral auxiliary to access the asymmetric synthesis of (−)-serotobenine (Scheme 4b).^21^

Recently, Yu and Wang realized the intramolecular C–H insertion step of the enantioselective synthesis of (+)-lithospermic acid by combining Davies's catalyst Rh_2_(*S*-DOSP)_4_ and Fukuyama's chiral auxiliary, achieving good yield and diastereoselectivity (Scheme 4c).^22^

**Scheme 5:**
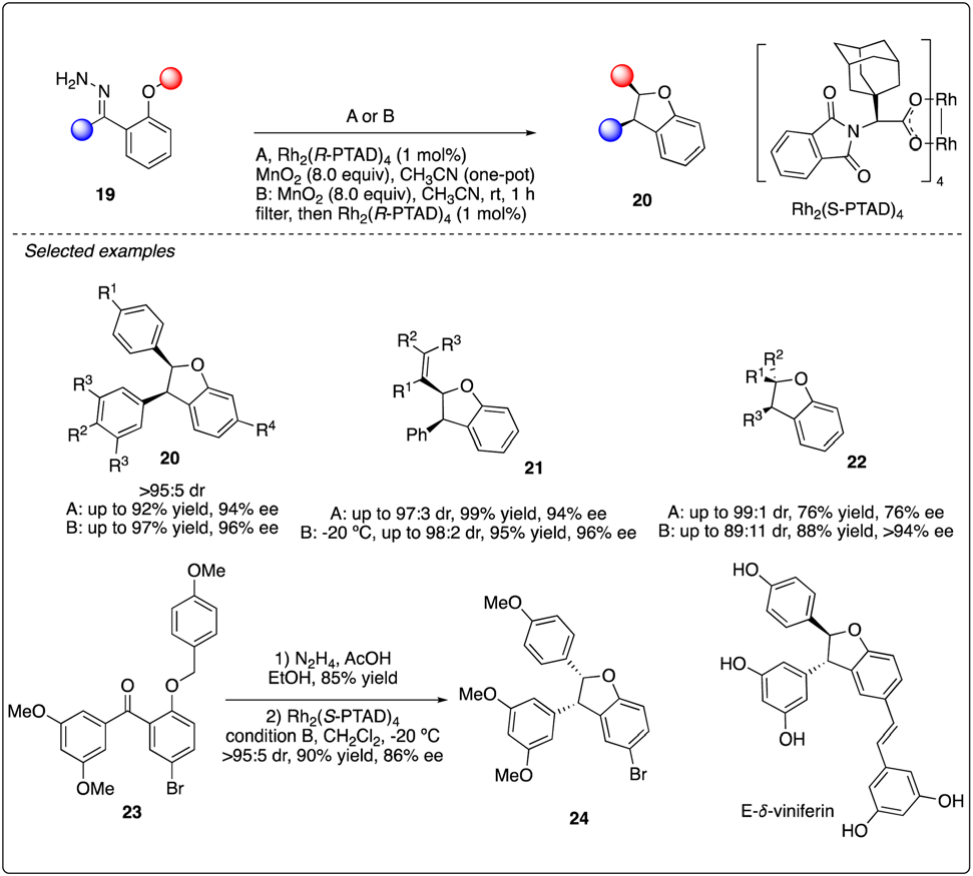
Enantioselective intramolecular C–H insertion reactions of donor–donor carbenoid and key step in total synthesis of *E-δ*-viniferin.

Additionally, Shaw reported Rh_2_(*R*-PTAD)_4_-catalyzed enantioselective intramolecular C–H insertion reactions of donor–donor carbenoid with very high stereoselectivity and good to excellent enantioselectivity (Scheme 5). They developed two unique reaction conditions to achieve the oxidation and insertion reactions step by step or in one-pot from hydrazone 19. This methodology was successfully applied to the asymmetric synthesis of *E-δ*-viniferin.^23^

**Scheme 6:**
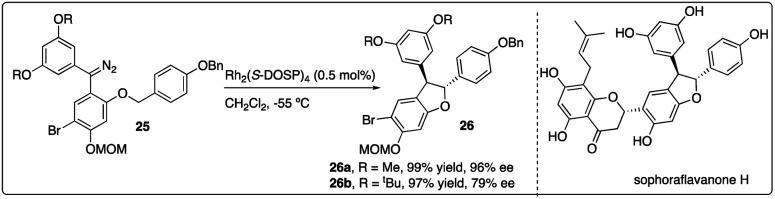
Key step in total synthesis of sophoraflavanone H by Kan group.

Very recently, Kan and colleagues constructed the dihydrobenzofuran ring 26 of natural product sophoraflavanone H *via* Rh_2_(*S*-DOSP)_4_-catalyzed asymmetric C–H insertion reaction of donor–donor carbinoid 25, achieving excellent yield and moderate enantioselectivity. Interestingly, the enantioselectivity of the dihydrobenzofuran 26 could be enhanced by introducing a methyl ether, albeit the removal of which posed challenges (Scheme 6).^24^

### 2.2 Synthesis of chromanones and pyran

**Scheme 7:**
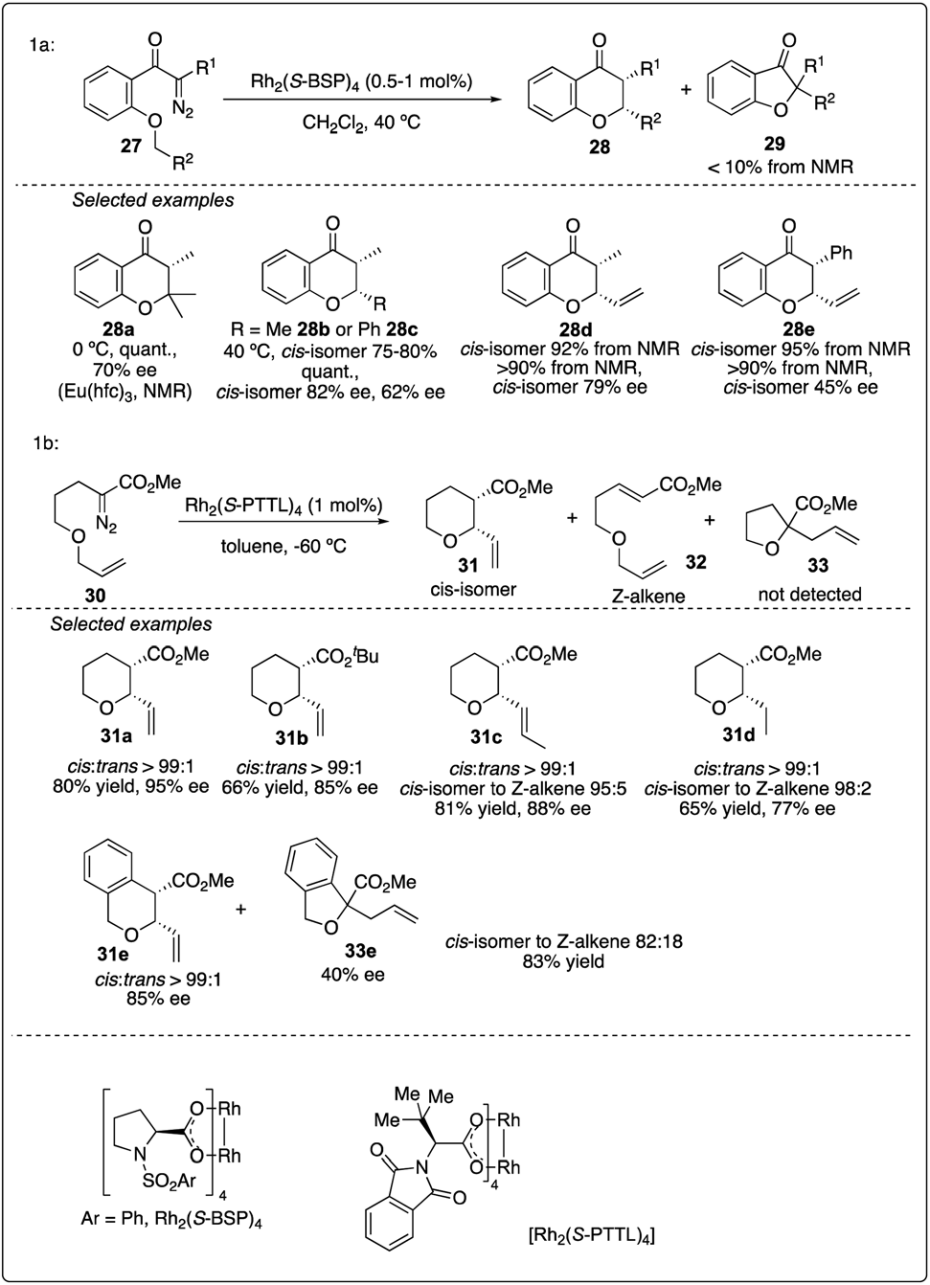
Chromanones and pyran synthesis *via* enantioselectivity intramolecular C–H insertion reactions.

The efficiency of asymmetric intramolecular carbenoid C–H insertion has been demonstrated to preferentially produce five and six-membered rings. In 1992, McKervey and Ye reported Rh_2_(*S*-BSP)_4_-catalyzed asymmetric C–H insertion reaction of ketocarbenoids, leading to the formation of chromanones 28 in excellent yields and good ee values (Scheme 7a)^25^ In 2015, Hashimoto and colleagues achieved the Rh_2_(*S*-PTTL)_4_-catalyzed asymmetric 1,6-C–H insertion reaction of α-diazo esters 30 by slightly modifying the substrate structure. This method was preferred over Z-alkene formation or tandem ylide formation-rearrangement and 1,2-hydride shift product (Scheme 7b).^26^

### 2.3 Synthesis of β-lactones

**Scheme 8:**
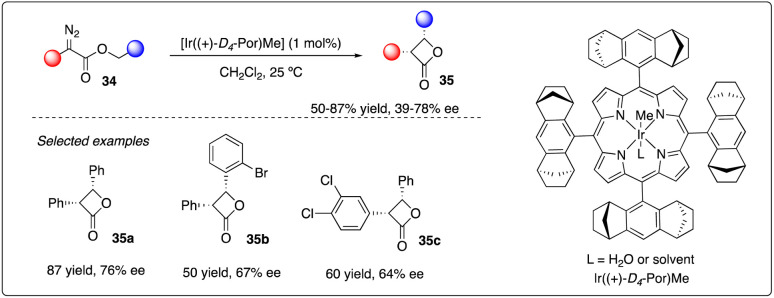
Synthesis of β-lactones *via* iridium catalyzed asymmetric C–H insertion reactions by Che group.

Four-membered rings like β-lactones are common yet unique structural motifs in natural products and pharmaceuticals, posing a challenge but accessible *via* intramolecular C–H carbene insertion. In 2013, Che and coworkers reported the use of [Ir((+)-*D*4-Por)Me], supported by *D*4-Halterman porphyrin ligand, as iridium catalysis. They successfully achieved enantioselective intramolecular carbene insertion into C–H bonds of α-diazoesters 34, yielding a series of aromatic substituted *cis*-β-lactones 35 with good stereoselectivity and enantioselectivities (Scheme 8).^27^

**Scheme 9:**
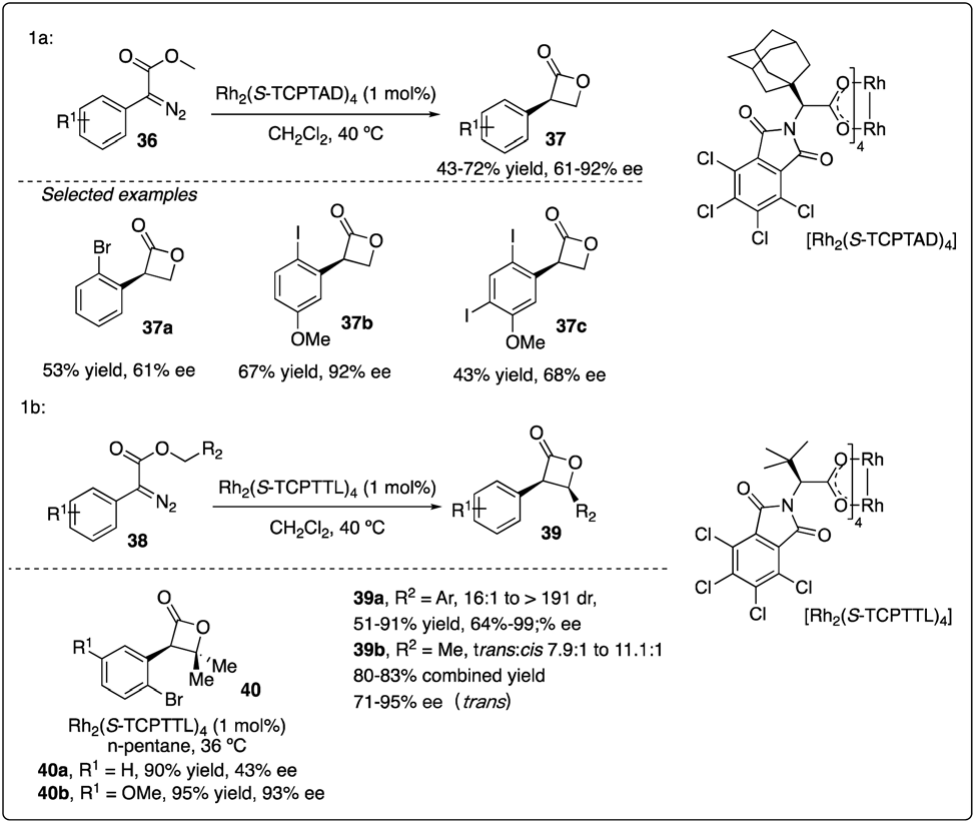
Synthesis of β-lactones *via* rhodium catalyzed asymmetric C–H insertion reactions by Davies group.

**Scheme 10:**
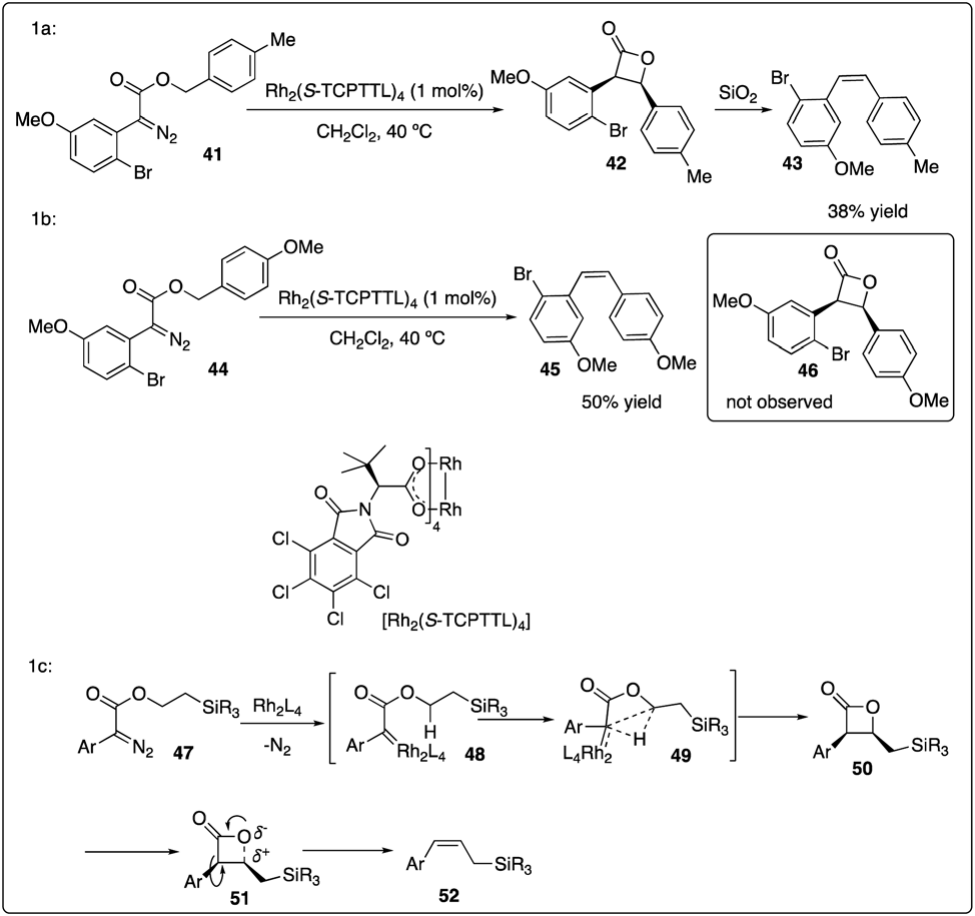
Control reactions and proposal mechanism for Z-product formation by Davies group.

Davies and colleagues discovered that the *ortho*-substituent (such as halo and trifluoromethyl) on aryldiazoacetate 36 could interfere with intermolecular C–H insertion, thereby enhancing the formation of β-lactone 37. Firstly, they developed asymmetric methyl C–H insertion catalyzed by Rh_2_(*S*-TCPTAD)_4_, achieving good to excellent enantioselectivities. Secondly, they switched to Rh_2_(*S*-TCPTTL)_4_ to modify the *cis*/*trans* selectivity of methylene C–H insertion (Scheme 9).^28^ Interestingly, when ethyl aryldiazoacetate 38b was employed as substrates, *trans*-formed product 39b was obtained with good to excellent enantioselectivity. However, when electron-donating methyl or methoxyl group (Scheme 10a and b) were introduced to the *para*-position of the benzyl ester, the desired C–H insertion product 42 was unstable to undergo CO_2_ extrusion, similar to previous findings reported by the same group (Scheme 10c). They proposed the mechanism for Z-product formation, suggesting that the silicon could direct the C–H insertion away from the more sterically hindered *α*-position and facilitate the polarization of the lactone C–O bond.^29^

## 3. Intermolecular α-C–H bond carbenoid insertion of ether

**Scheme 11:**
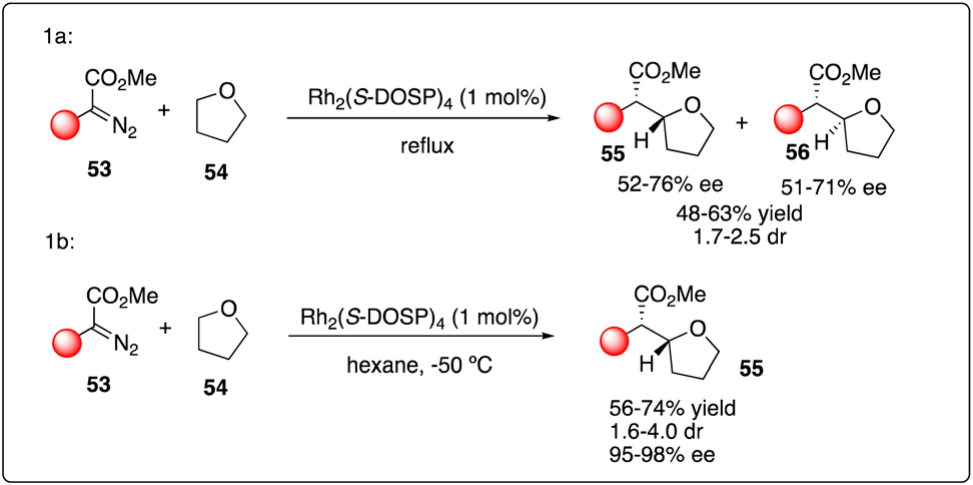
Enantioselective intermolecular C–H insertion reactions of donor–accepter carbenoid and THF by Davies group.

The challenge of achieving asymmetric intermolecular carbenoid C–H insertion persisted until the emergence of a robust catalyst system system capable of overcoming these major obstacles in organic synthesis. Based upon their previous study on Rh_2_(*S*-DOSP)_4_-catalyzed asymmetric cyclopropanations with diazoacetates, Davies and Hansen proposed utilizing the decomposition of aryldiazoacetates 53 by Rh_2_(*S*-DOSP)_4_ to enable asymmetric intermolecular C–H insertions. Employing this strategy, they successfully realized the C–H insertion of aryldiazoacetates 53 with tetrahydrofuran 54 as a carbenoid trap, yielding a series of products with moderate dr values and good enantioselectivity (Scheme 11a).^30^ Davies and Hansen also proposed the asymmetric induction model by Rh_2_(*S*-DOSP)_4_ to predict the configuration in the product. Subsequently, they optimized the reaction conditions,^31^ discovering that the degassing of the solvent significantly increased the reactivity and the enantioselectivity. Furthermore, they achieved further improvement in enantioselectivity by employing nonpolar solvents such as THF (2 equiv.) in hexane, highlighting the remarkable selectivity of the carbenoid in the presence of hexane (Scheme 11b). Additionally, they investigated the kinetic isotope effect for the C–H insertion process through competition experiments between d8-tetrahydrofuran and tetrahydrofuran.

**Scheme 12:**
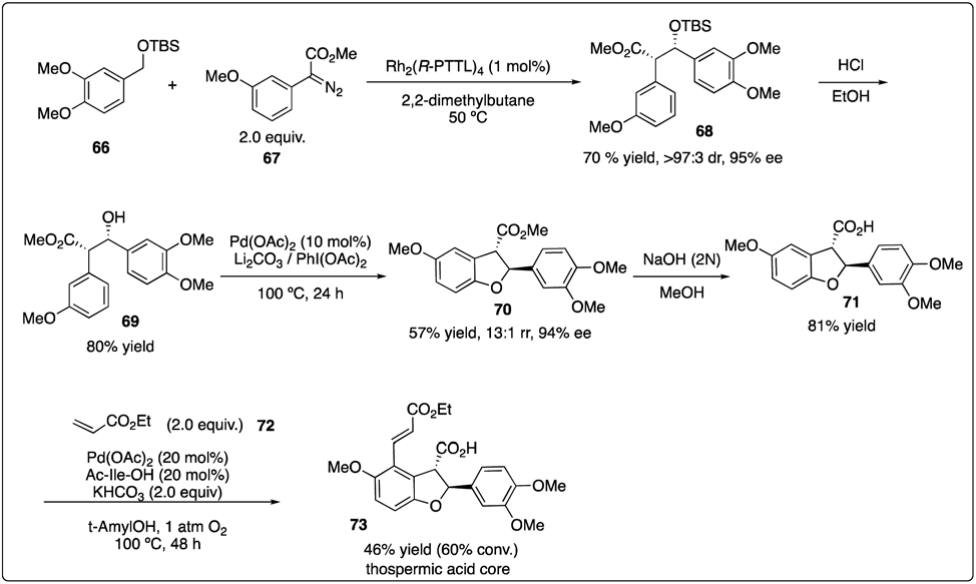
Enantioselective intermolecular C–H insertion reaction for the synthesis of thospermic acid core by Davies and Yu group.

Subsequently, they delved into the comprehensive chemistry of rhodium carbenoid-induced C–H insertion of silyl ethers 56 to synthesize silyl-protected β-hydroxy esters 58 with excellent regio-, diastereo-, and enantioselectivity (Scheme 12a).^32^ They particular emphasized the critical requirement for selectivity by utilizing donor/acceptor-substituted carbenoids 57 and catalytic rhodium prolinate complex Rh_2_(*S*-DOSP)_4_. Additionally, they discovered that the C–H insertion reaction is facilitated with the *trans*-allyl silyl ether and summarized the general reactivity trends (Scheme 12b). They elucidated that a silyl allyl ether is highly activated for C–H insertion, while acetoxyl group exerts a deactivating effect presumably due to its electron-withdrawing nature. Despite consistently low enantioselectivity observed in the C–H insertion of benzyl silyl ethers 59 by Rh_2_(*S*-DOSP)_4_ (10–35% ee), and moderately high enantioselectivity achieved through chiral auxiliary mediated C–H functionalization reactions using ethyl (*S*)-lactate-based aryldiazoacetate (79–88% de, 68–85% ee), they ultimately attained the best results (91–95% de, 95–98% ee) by employing Hashimoto's Rh_2_(*S*-PTTL)_4_ catalyst.^33^

Recently, Davies and Sigman and their coworkers discovered that the site- and stereoselective C–H functionalization at *α*, *γ*, *δ* and even more distal positions to the siloxy ethers could be achieved by leveraging the preferences of Rh_2_(*R*-TCPTAD)_4_ and Rh_2_(*S*-2-Cl-5-BrTPCP)_4_ catalyst, yielding products with good to excellent enantioselectivity (Scheme 12c).^34^ Additionally, they developed a machine learning classification model to predict the major C–H functionalization site based on catalyst propensity and substrate electronics.

**Scheme 13:**
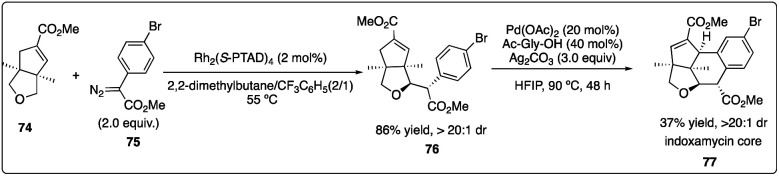
Enantioselective intermolecular C–H insertion reaction for the synthesis of indoxamycin core by Sorensen group.

**Scheme 14:**
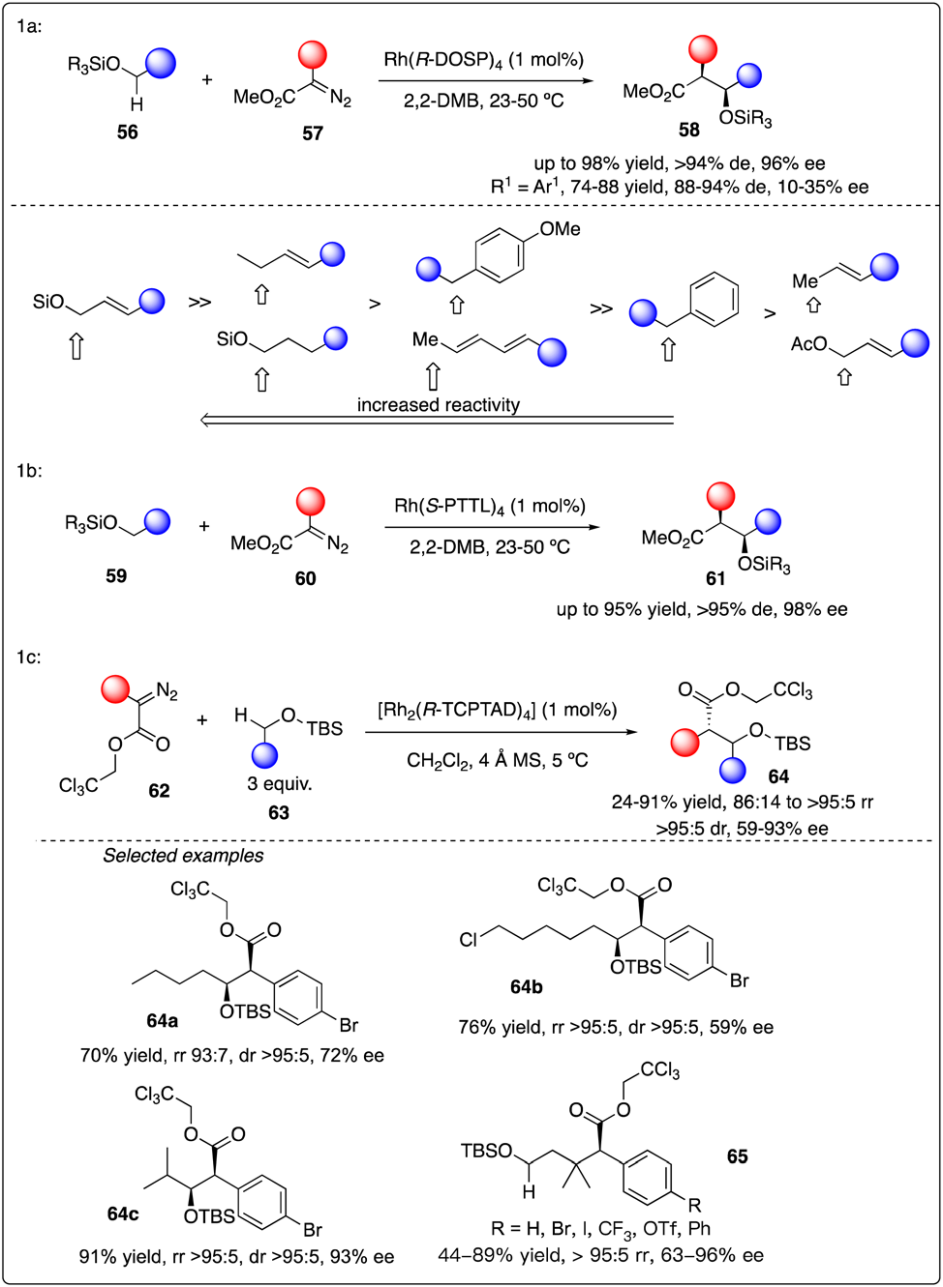
Enantioselective intermolecular C–H insertion reactions of donor–accepter carbenoid and silyl ether by Davies group.

In 2013, Davies and Yu and their coworkers achieved a highly regio-, diastereo-, and enantioselective synthesis of dihydrobenzofurans 73 (the core of the thospermic acid family) involving three distinct C–H functionalization steps. Particularly noteworthy was a rhodium-catalyzed enantioselective intermolecular C–H insertion reaction (Scheme 13).^35*a*^ Later, in a collaboration between the Sorensen and Yu groups with the Davies groups, another two sequential C–H functionalization reactions were described for the synthesis of the indoxamycin family core 77 with good yield and highly regio- and di stereoselectivity (Scheme 14).^35*b*^

**Scheme 15:**
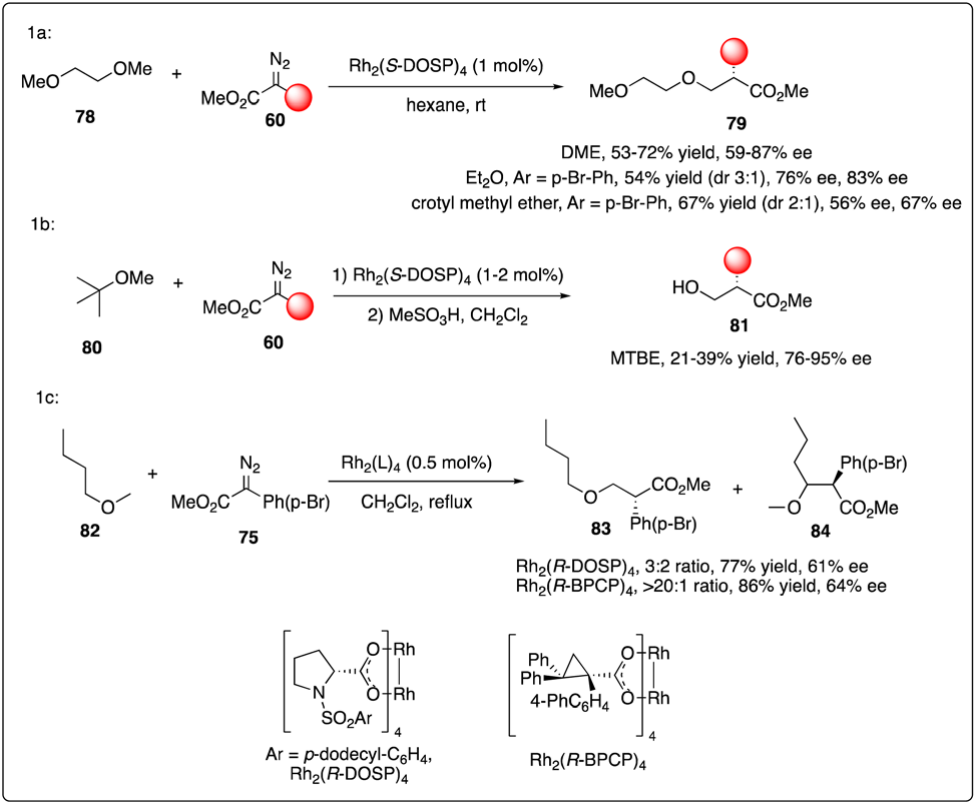
Enantioselective intermolecular C–H insertion reactions of donor–accepter carbenoid and primary methine by Davies group.

The electronic stabilization of the transition state during C–H activation favored α to alkoxy or silyloxy groups in a concerted non-synchronous manner, as demonstrated with excellent enantioselectivity and regioselectivity results. However, the Davies group observed a dramatical site selectivity when alkyl ethers like DME 78, Et_2_O, and unsymmetric ethers were used.^36*a*^ In comparison to a mixture obtained with Rh_2_(*R*-DOSP)_4_ catalyst, the Rh_2_(*R*-BPCP)_4_ catalyzed C–H carbenoid insertion reaction selectively targeted the primary C–H bond with moderate enantioselectivity (Scheme 15).^36*b*^

**Scheme 16:**
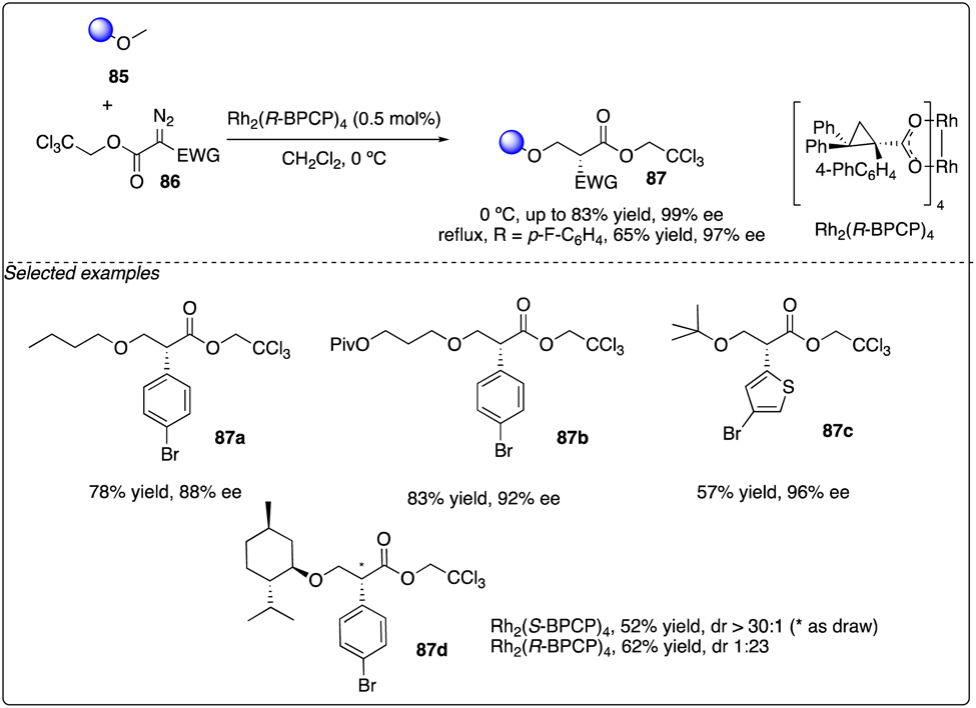
Enantioselective intermolecular C–H insertion reactions of donor–accepter (TCE) carbenoid and primary methine by Davies group.

Later, Davies and Guptill optimized the aforementioned C–H insertion reaction of diazo compounds 86 with a 2,2,2-trichloroethyl (TCE) ester as the acceptor group. They discovered that the TCE ester could enhance reactivity and improve the levels of regioselectivity and enantioselectivity between primary and secondary C–H bonds (Scheme 16).^37^

**Scheme 17:**
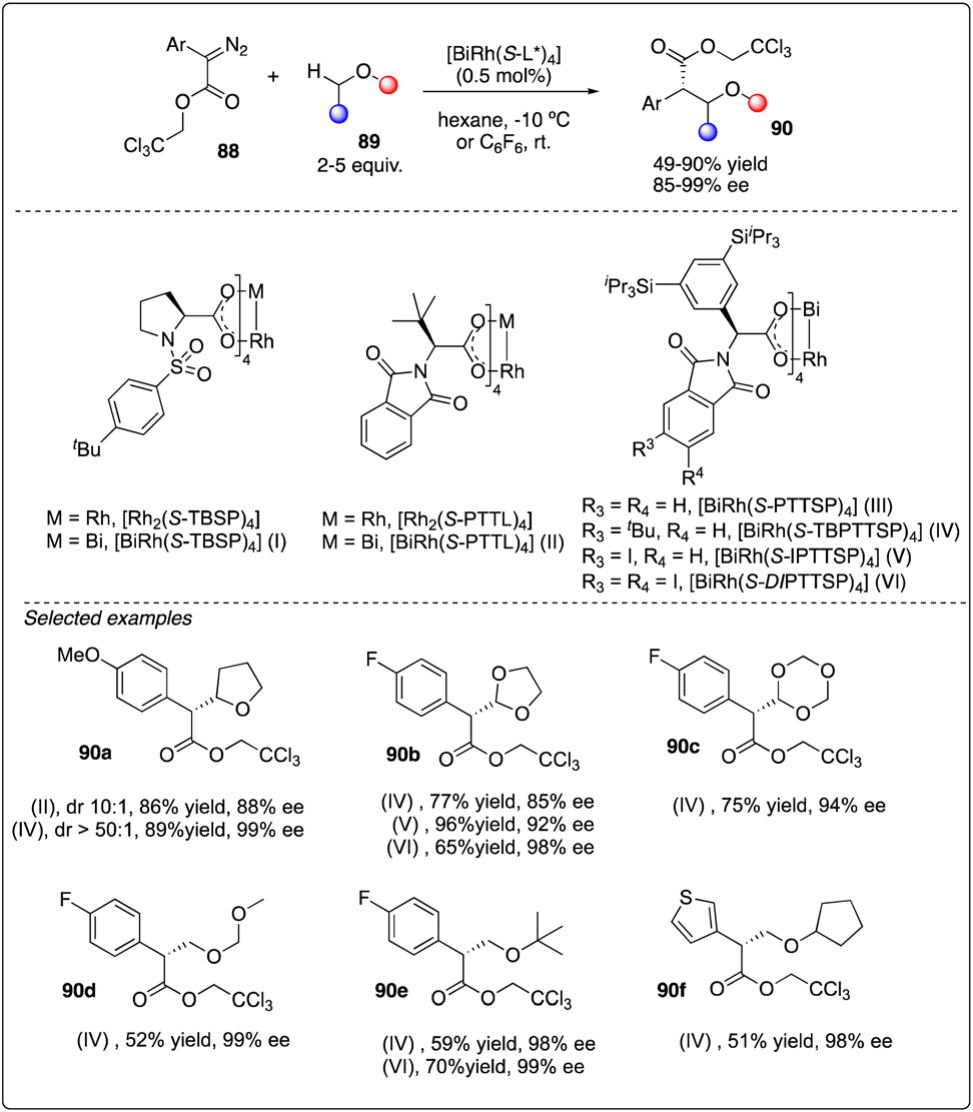
Enantioselective intermolecular C–H insertion reactions of donor–accepter carbenoid with chiral [RhBi] catalyst by Fürstner group.

Recently, Davies, Musaev and Berry and their coworkers reported the first chiral [RhBi] paddlewheel catalyst, [BiRh(*S*-TBSP)_4_], and applied them to asymmetric cyclopropanation and C–H functionalization.^38^ Later, Fürstner and his coworkers designed a new type of [RhBi] paddlewheel catalysts^39^ and found it to be exceedingly effective in site-selective C–H functionalization with ethers (Scheme 17).^40^ Their rationale catalyst design involved using phenyl rings carrying lateral –Si(iPr)_3_ substituents to replace the *tert*-butyl residues, facilitating a large number of attractive interligand London dispersion interactions.^41^

**Scheme 18:**
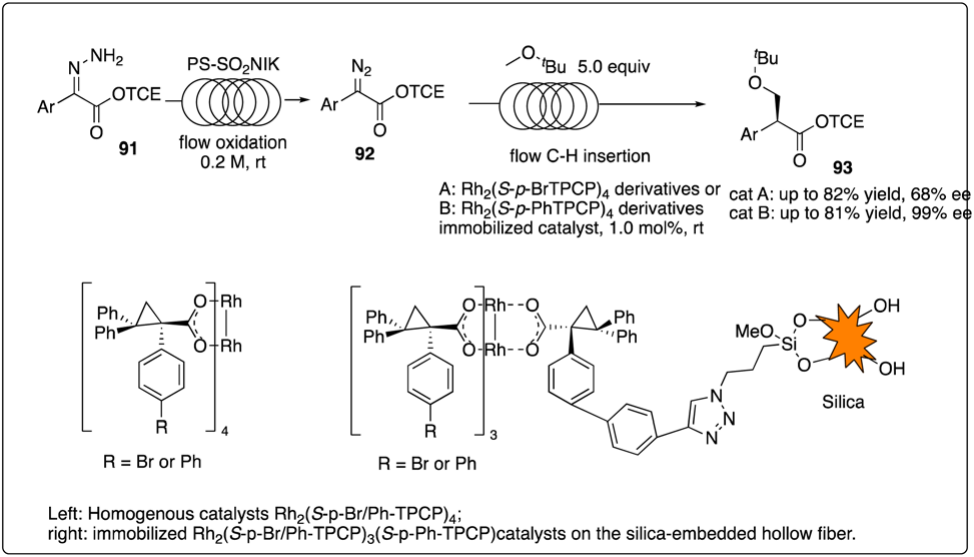
Enantioselective intermolecular C–H insertion reactions with a scalable flow reactor by Davies and Jones group.

Davies and Jones and their coworkers developed a scalable flow reactor for enantioselective and regioselective rhodium C–H carbene insertion reactions (Scheme 18).^42^ Comparing to conventional batch reactors, the yield, site-selectivity and enantioselectivity of the product in this immobilized dirhodium hollow-fiber flow reactor were similar but more effective, scalable, recyclable.

**Scheme 19:**
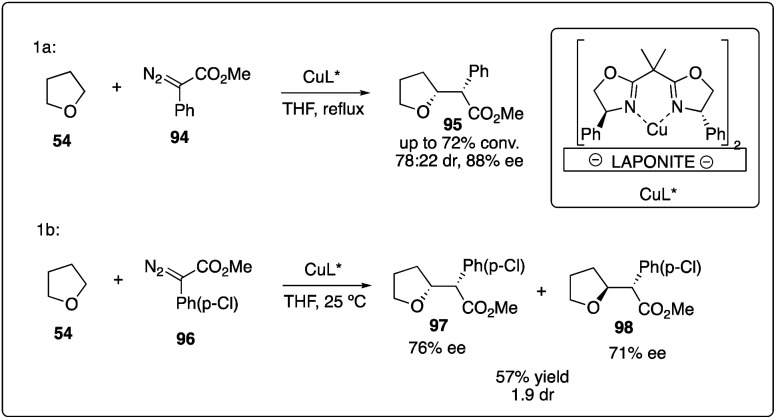
Enantioselective intermolecular C–H insertion reactions of donor–accepter carbenoid and THF catalyzed by copper complex.

Copper complex was the first transition metal catalyst proposed to promote the decompositions of carbenoids.^4^ However, it was only recently that the asymmetric version for the α-C–H activation/carbenoid insertion of ethers was realized. In 2007, Fraile, Mayoral and coworkers reported an immobilized box–Cu complex catalyzed asymmetric insertion of a carbene into C–H bonds of THF with good enantioselectivity (Scheme 19a).^43^ This bis(oxazoline)-copper complex supported by Laponite could be recovered and reused with good reactivity and enantioselectivity control, demonstrating catalytic stability over three more cycles. Comparing with homogeneous box–copper complexes for carbene insertion reactions, the immobilized copper complexes could achieve higher chemoselectivities and stereoselectivities than normally used rhodium catalyst (Scheme 19b).^44^

**Scheme 20:**
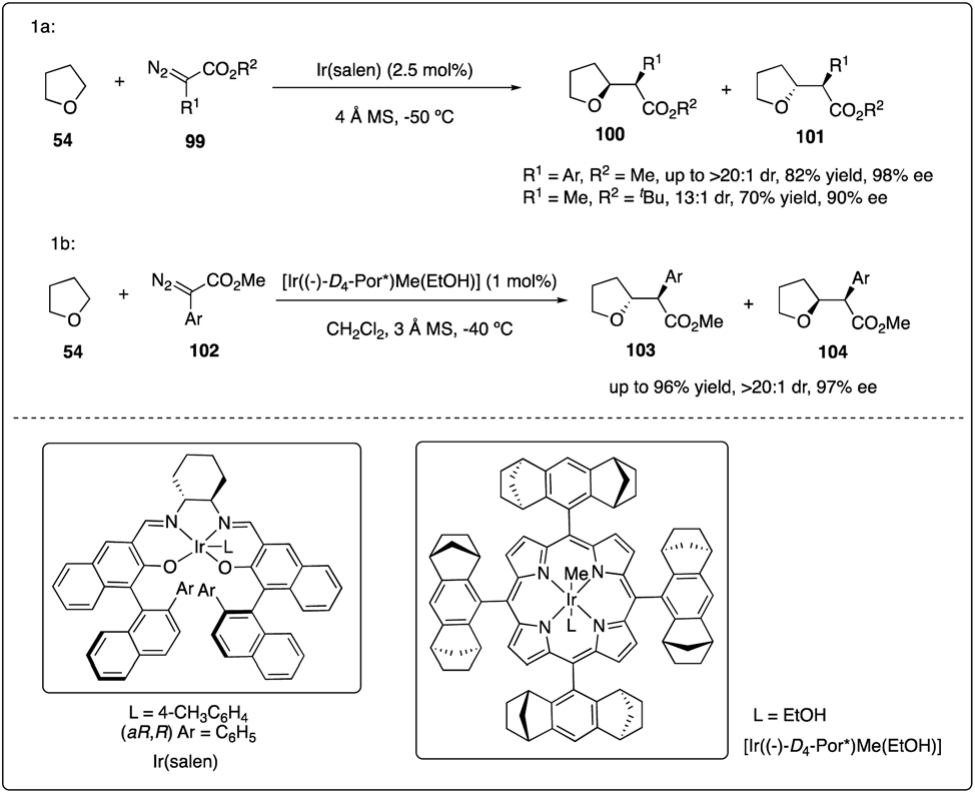
Enantioselective intermolecular C–H insertion reactions of donor–accepter carbenoid and THF catalysed by iridium complex.

Katsuki and Suematsu discovered the iridium(iii)-salen complexes show promising results in asymmetric carbenoid C–H insertion reactions (Scheme 20a). All the aryldiazoacetates 99 were well tolerated with good to excellent diastereoselectivity and enantioselectivity. They also achieved the first example of asymmetric intermolecular insertion with alkyl-substituted diazoacetate.^45^ Later, Che and coworkers reported chiral iridium porphyrin catalyzed asymmetric C–H carbene C–H insertion reactions with good to excellent diastereoselectivity, enantioselectivity and product turnovers (Scheme 20b).^46^

## 4. Summary and outlook

Catalytic asymmetric α-C–H bond functionalization of ether *via* carbenoid insertion has made enormous progress over the past decade. Effective asymmetric catalytic systems, including various chiral metal catalysts, have already been established for the enantioselective carbenoid insertion into the C–H bond adjacent to the oxygen atom in a variety of structurally diverse ether substrates. This has led to the generation of various enantioenriched highly-functionalized oxygen-containing structural motifs, enabling corresponding applications in asymmetric synthesis of bioactive natural products.

Despite these elegant advancements, challenges still remain and further exploration of catalytic enantioselective insertion of carbenoids into α-C–H bond functionalization of ethers is highly in demand. Currently, rhodium based asymmetric catalytic systems are the most commonly employed and well-studied, however, asymmetric catalytic systems based on more readily available transition metals, such as copper, iron, and cobalt, which might enhance the value of asymmetric C–H functionalization reactions, are underexplored and should draw more attention from the chemistry community. In addition, researches in this area is likely to focus on novel ligand families design and development to explore new type of substrates and transformations with a high level of chemo-, regio- and/or stereo-selectivities. Moreover, the discovery and further development of carbenoid insertion transformations with the *in situ* generated diazo substrates from more accessible precursors, like hydrazones, would greatly promote the practicality and versatility of current asymmetric C–H functionalization methodologies. Despite these challenges, we believe that catalytic asymmetric carbenoid insertion into α-C–H bond of ether will undoubtedly witness improvement and meet the requirements for more practical applications in the synthesis of pharmaceuticals or natural products in the future.

## Conflicts of interest

There are no conflicts to declare.

## Supplementary Material
